# Barriers and facilitators of implementing interventions to improve appropriate antibiotic use in low- and middle-income countries: a systematic review based on the Consolidated Framework for Implementation Research

**DOI:** 10.1186/s13012-022-01209-4

**Published:** 2022-05-12

**Authors:** Shishi Wu, Elias Tannous, Victoria Haldane, Moriah E. Ellen, Xiaolin Wei

**Affiliations:** 1 Dalla Lana School of Public Health, University of Toronto, Toronto, Canada; 2 Faculty of Health Sciences, Department of Clinical Biochemistry and Pharmacology, Ben Gurion University of the Negev, Beer-Sheva, Israel; 3 Pharmacy services, Hillel Yaffe Medical Center, Hadera, Israel; 4 Institute of Health Policy, Management, and Evaluation, Dalla Lana School of Public Health, University of Toronto, Toronto, Canada; 5 Department of Health Policy and Management, Guilford Glazer Faculty of Business and Management and Faculty of Health Sciences, Ben-Gurion University of the Negev, Beer-Sheva, Israel

**Keywords:** Antimicrobial resistance, Implementation science, Barriers and facilitators, Rational antibiotic use

## Abstract

**Background:**

Behavior change interventions that aim to improve rational antibiotic use in prescribers and users have been widely conducted in both high- and LMICs. However, currently, no review has systematically examined challenges unique to LMICs and offered insights into the underlying contextual factors that influence these interventions. We adopted an implementation research perspective to systematically synthesize the implementation barriers and facilitators in LMICs.

**Methods:**

We conducted literature searches in five electronic databases and identified studies that involved the implementation of behavior change interventions to improve appropriate antibiotic use in prescribers and users in LMICs and reported implementation barriers and facilitators. Behavior change interventions were defined using the behavior change wheel, and the coding and synthesis of barriers and facilitators were guided by the Consolidated Framework for Implementation Research (CFIR).

**Results:**

We identified 52 eligible studies, with the majority targeting prescribers practicing at tertiary facilities (*N*=39, 75%). The most commonly reported factors influencing implementation were found in the inner setting domain of the CFIR framework, particularly related to constraints in resources and the infrastructure of the facilities where interventions were implemented. Barriers related to the external policy environment (e.g., lack of national initiatives and policies on antibiotic use), and individual characteristics of target populations (e.g., reluctance to change prescribing behaviors) were also common, as well as facilitators related to intervention characteristics (e.g., embedding interventions in routine practice) and process (e.g., stakeholder engagement). We also provided insights into the interrelationships between these factors and the underlying causes contributing to the implementation challenges in LMICs.

**Conclusion:**

We presented a comprehensive overview of the barriers and facilitators of implementing behavior change interventions to promote rational antibiotic use in LMICs. Our findings suggest that facilitating the implementation of interventions to improve rational antibiotic use needs comprehensive efforts to address challenges at policy, organizational, and implementation levels. Specific strategies include (1) strengthening political commitment to prompt mobilization of domestic resources and formulation of a sustainable national strategy on AMR, (2) improving the infrastructure of health facilities that allow prescribers to make evidence-based clinical decisions, and (3) engaging local stakeholders to improve their buy-in and facilitate contextualizing interventions.

**Trial registration:**

PROSPERO: CRD42021252715.

**Supplementary Information:**

The online version contains supplementary material available at 10.1186/s13012-022-01209-4.

Contributions to the literature

- Behavior change interventions are widely used to address inappropriate antibiotic use—a major driver of AMR in LMICs. Our review is the first to give a comprehensive overview of the barriers and facilitators of implementing behavior change interventions to promote rational antibiotic use in LMICs.
- The findings of this review are helpful for researchers and implementers to directly assess the challenges posed by context and respond by contextualizing their interventions when developing similar interventions.
- We provided insights into the interrelationships between the commonly reported barriers and facilitators, and the underlying factors contributing to the implementation challenges in LMICs, thus recommending a comprehensive approach to addressing these challenges at policy, organizational, and implementation levels.

## Background

Antibiotics are critical for treating infections caused by bacteria [1]. However, the development of drug resistance makes common infections difficult to treat, thus increasing the risk of disease spread, severe illness, and death. As drug-resistant pathogens emerge and spread, the development of new antibiotics is lagging. In 2019, the World Health Organization (WHO) identified 32 antibiotics in clinical development that address the WHO list of priority pathogens, of which only six were classified as innovative [2]. With accelerating drug resistance, and limited effective agents for treating infections, we face a future with rising mortality due to infection and where the safety of conducting life-saving medical procedures, such as surgery and organ transplantation, will be increasingly threatened [2].

As such, antimicrobial resistance (AMR) poses a major threat to global health. Low- and middle-income countries (LMICs) are especially vulnerable and threatened by the increasing burden of AMR owing to a high incidence of infectious diseases [3, 4], weak regulatory system, inappropriate prescription and use of antibiotics, gaps in diagnostic testing and surveillance, and frequent use of antibiotics in livestock production [5]. Inappropriate antibiotic use is particularly salient and prevalent in LMICs [6]. For prescribers, inappropriate antibiotic use includes “unnecessary prescribing of antibiotics and also when an antibiotic is needed but the wrong antibiotic is prescribed or the wrong dose is given or the antibiotic is prescribed for the wrong length of time” [7]. For consumers, it involves behaviors such as using antibiotics without prescriptions, not adhering to instructions from providers, and not completing treatment courses [8]. Previous studies have identified a wide range of drivers of inappropriate antibiotic use in LMICs, including lack of knowledge among antibiotic users [4, 9, 10], inadequate training and supervision provided for health workers [11–13], prescribers’ habit [9, 14], pharmaceutical promotion [13, 14], lack of diagnostic tools [14], economic incentives for suppliers and prescribers [14, 15], and the influence of peers and community members [15]. As such, behavior change interventions tackling drivers of inappropriate antibiotic use are central to the operation of the WHO Global Action Plan (GAP) for AMR and achieving the milestones of combating AMR set by individual countries [16]. Behavior change interventions have been defined by Michie et al. as coordinated sets of activities designed to change specified behavior patterns [17].

Existing reviews reported that implementation of behavior change interventions, such as audit and feedback [18, 19], lectures, and enforcing guidelines [20], were effective in promoting appropriate prescribing practice among prescribers [21, 22]. In addition to interventions targeting prescribers, educational sessions, mass media campaigns, and distributing learning material at schools and households have been employed in many countries to improve the knowledge and appropriate behavior among users [23, 24]. However, while many empirical studies report implementation issues, the majority of existing systematic reviews focused on intervention outcomes and effectiveness without offering insights into challenges in the implementation process or elaborating on the context in which interventions are designed and conducted [24–26]. Apart from a previous narrative review of the challenges for antimicrobial stewardship programs (ASPs) in LMICs [27], currently, no review has systematically examined factors unique in LMICs that facilitate or undermine the implementation of interventions tackling inappropriate use of antibiotics in both prescribers and consumers.

Implementation research intends to understand why, in what context, and for whom interventions work in “real-world” settings [28]. There has been increasing recognition of the need for theory-based strategies to facilitate implementation and a growing interest in framework-based approaches to gain insights into factors contributing to the success of an intervention or a program [29]. This is because designing, adapting, implementing, and sustaining an intervention is inherently complex. Applying theories and frameworks that are developed from an implementation research perspective provides researchers and implementers a clear, consistent, and systematic way to build knowledge on what works and the underlying contextual factors contributing to its success [30].

In this study, we adopted an implementation research perspective to systematically synthesize the barriers and facilitators of implementing behavior change interventions that aim to improve appropriate antibiotic use in LMICs.

## Methods

### Conceptual framework

The Consolidated Framework for Implementation Research (CFIR) is composed of 39 constructs across five domains that influence the implementation process and outcomes. This framework has incorporated constructs from published theories and models into one comprehensive framework, thus providing a structured and systematic way to assess the context within which implementation occurs [31]. The application of this framework facilitates comparisons between studies that were conducted in different settings and allows a comprehensive synthesis of factors influencing intervention implementation at multiple levels. It also provides a foundation to develop multifaceted implementation strategies tailored to LMIC settings for improving the adoption, implementation, and sustainability of health interventions [32]. In this review, we used the CFIR framework to guide the analysis and synthesis of implementation barriers and facilitators reported in the reviewed studies.

We define behavior change interventions using the behavior change wheel proposed by Michie et al., which consists of a behavior system at the hub, encircled by intervention functions and policy categories [17]. The nine intervention functions include education, persuasion, incentivization, coercion, training, restriction, environmental restructuring, modeling, and enablement (Table 1). This framework guided the formulation of our search strategy and inclusion criteria and facilitated categorizing of the interventions described in the reviewed studies.

**Table 1:** Definitions of the nine intervention functions of the behavior change wheel

Intervention function	Definition
Education	Increasing knowledge or understanding.
Persuasion	Using communication to induce positive or negative feelings or stimulate action.
Incentivization	Creating expectation of reward.
Coercion	Creating expectation of punishment or cost.
Training	Improving skills.
Restriction	Using rules to reduce the opportunity to engage in the target behavior.
Environmental restructuring	Changing the physical or social context.
Modeling	Providing an example for people to aspire to or imitate.
Enablement	Increasing means or reducing barriers to increase capability or opportunity.

### Search strategy

The protocol of this review was registered in PROSPERO (CRD42021252715). The population of the review included healthcare providers who are qualified to prescribe antibiotics and users of antibiotics. The interventions of interest were those that included at least one of the nine intervention functions of the behavior change wheel with an aim of improving the rational use of antibiotics in LMICs. Since we were interested in implementation barriers and facilitators reported in studies, we did not restrict studies by study design or implementation outcomes.

We conducted literature searches in five electronic databases, including MEDLINE, EMBASE, CINAHL, Cochrane Library, and Web of Science and obtained the first 100 titles from Google Scholar. The search was run-up to May 13, 2021. A search strategy consisting of key words of the research scope, target populations, intervention types, and settings was developed with the assistance of a librarian and used to retrieve relevant articles (see Additional file 1).

### Study selection

A two-stage screening process was conducted to select eligible studies that met the inclusion criteria, as shown in Table 2. In the first stage of screening, two researchers (SW and VH) independently reviewed the titles and abstracts of the retrieved studies. Results from both researchers were compared, and titles for which an abstract was not available or for which either of the reviewers’ suggested inclusion were put forward for subsequent full-text review (performed by SW and ET) as part of the second stage of eligibility screening. As stated in the inclusion and exclusion criteria, we only included studies that reported implementation facilitators and barriers from primary data collection or based on authors’ own reflections after implementing behavior change interventions (e.g., implementation barriers and facilitators experienced and reported by authors in studies that described or evaluated behavior change interventions). Commentary pieces or barriers and facilitators identified from literature review without actual implementation of interventions were excluded. Any disagreement occurring during the screening process was resolved via consensus. Articles that could not be obtained through online databases and library searches were excluded in the final analysis.

**Table 2:** Inclusion and exclusion criteria

Inclusion criteria	• The population of the study should be healthcare providers who are qualified to prescribe antibiotics and users of antibiotics • The aim of the intervention is to improve appropriate prescription of antibiotics by healthcare providers or rational use of antibiotics among the consumers. Interventions should include at least one of the intervention functions in the behavior change wheel. • Studies describing the designing, implementation, or evaluation (including outcome evaluation, process evaluation, and barriers analysis) of a behavior change intervention • Limit to studies that were conducted in LMICs • No restrictions in publication year • Limit to human research • Studies that reported implementation facilitators and barriers from primary data collection or based on authors’ own reflections after implementing behavior change interventions
Exclusion criteria	• Editorials, commentary pieces, and systematic reviews • Animal studies • Studies that assess compliance to existing guidelines • Interventions targeting medical students • Study protocols and studies in which implementation facilitators and barriers were not reported. • Articles not in English or Chinese

### Quality appraisal

Since we expected to include studies with a wide range of methodologies, the quality of included studies was assessed using the Mixed Methods Appraisal Tool (Version 2018), which appraises the methodological quality of five categories of studies: qualitative research, randomized controlled trials, non-randomized studies, quantitative descriptive studies, and mixed methods studies [33]. Each category of studies was assessed against five criteria and each criterion was rated by reviewers with “Yes”, “No”, or “Can’t tell”. Two researchers (SW and ET) performed a quality appraisal of the included studies independently.

### Data extraction and synthesis

Data extraction was completed by two researchers (SW and ET) using a predesigned excel sheet. We extracted information, including authors, publication year, country of study, study aim, study design, setting, target population, and description of the intervention reported in the study. Original text on implementation barriers and facilitators was directly extracted from the included studies. We categorized the interventions that were implemented and described in each study based on the definitions of the intervention functions provided in the behavior change wheel [17]. Interventions or programs that involved multiple components or delivered through different methods were assigned to multiple categories. Analysis of qualitative data on implementation facilitators and barriers was conducted by two reviewers (SW and ET) using a deductive coding approach and through an iterative process. Both reviewers coded the first five articles to the constructs of the CFIR framework using a codebook provided on the CFIR website [34]. The coding results were compared between the two reviewers, and any disagreement or modifications to construct definitions were discussed among the reviewers until a consensus was reached. A modified working codebook was adapted and used to guide the analysis of the remaining articles (Additional file 2). A review of constructs and supporting data following coding was conducted (SW). Inter-rater reliability was not calculated.

We first tabulated and conducted a narrative synthesis of the general characteristics of the included studies, then summarized the common implementation barriers and facilitators by the five domains of the CFIR framework. Additionally, we examined and mapped the relationships between the constructs within and across the domains of the CFIR framework based on the information reported in the primary studies. We completed the review in accordance with the Preferred Reporting for Systematic Reviews and Meta-Analyses (PRISMA) statement and checklist (Additional file 6).

## Results

### Search results and included studies

**Fig. 1:**
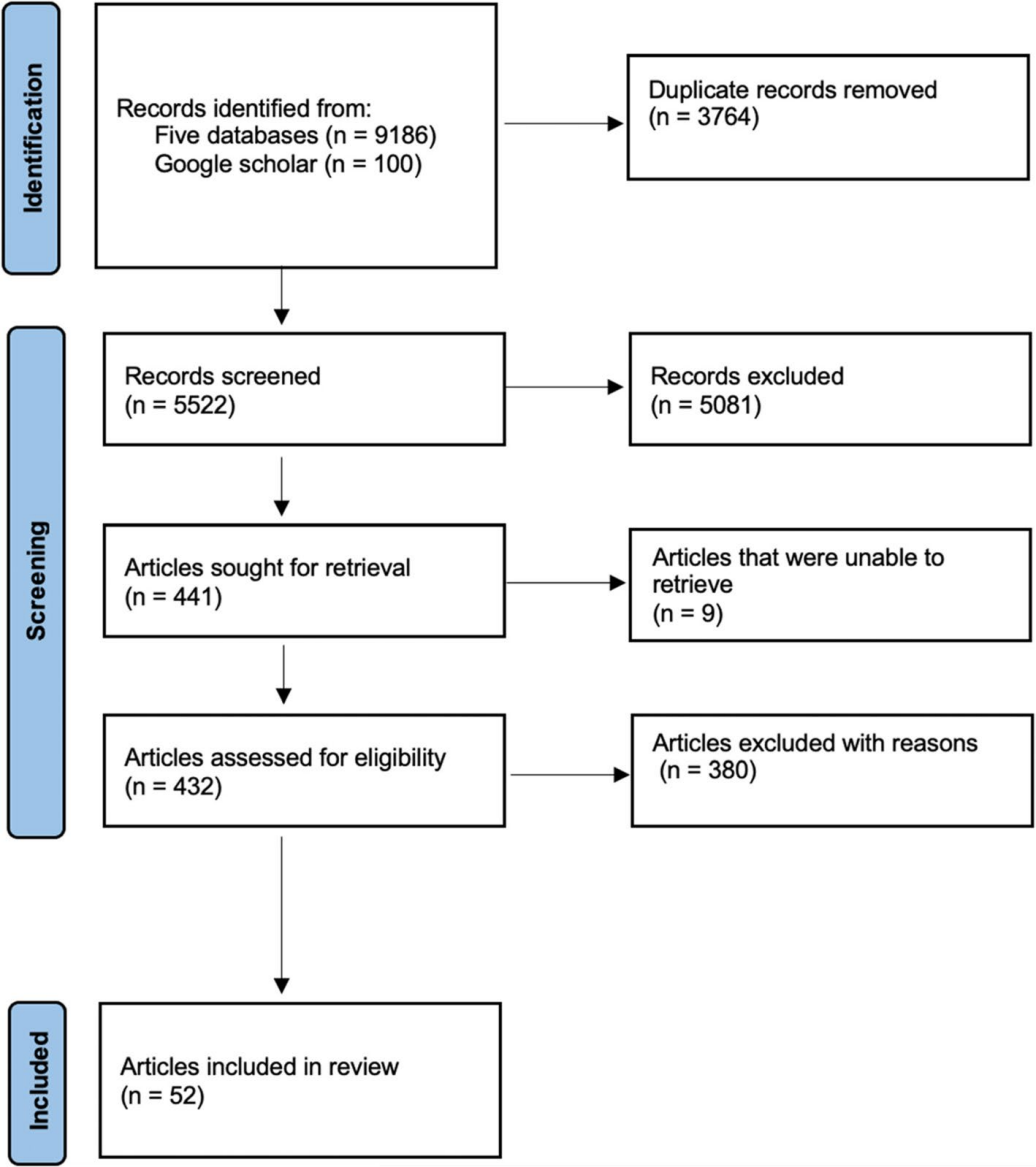
PRISMA flow chart

We retrieved a total of 9186 citations from electronic databases and 100 from Google Scholar (Fig. 1). After removing duplicates, we screened titles and abstracts of 5522 publications, of which 441 went through full-text review and 52 were included in the final analysis. The majority were excluded for not explicitly reporting implementation barriers and facilitators.

### Study characteristics and quality appraisal

Table 3 summarizes the characteristics of the 52 articles included in the review. Countries were classified based on the World Bank’s 2019 country classification [35], and we found that most studies were conducted in Sub-Saharan Africa (*N*=17, 33%) and East Asia and Pacific (*N*=16, 31%). About 75% of the studies were implemented in tertiary hospitals, followed by primary care settings (*N*=13, 25%), while community-based interventions were very few (*N*=4, 8%). Nearly half of the studies described interventions for improving the prescribing practice of physicians only (*N*=25, 48%), whereas interventions targeting users (*N*= 2, 4%) or both providers and users (*N*=3, 6%) were rarely reported. Additionally, 42% of the studies (*N*=22) described interventions that involved multiple providers, such as physicians, surgeons, pharmacists, and nurses. In terms of intervention types, most studies employed enablement (e.g., audit and feedback; *N*=35, 67%), restriction (e.g., developing and enforcement of guidelines; *N*=31, 60%), and education (e.g., information sessions; *N*=27, 52%) interventions, with only few studies used persuasion (*N*=4, 8%) and modeling (*N*=1, 2%) interventions. Additionally, in more than half of the studies, interventions included two or three components. The most commonly applied study designs were pre-post (*N*=17, 55%) and cross-sectional (*N*=15, 29%), while very few studies used more robust designs such as randomized control trials (*N*=1, 2%) or a quasi-experimental design (*N*=1, 2%). About 13% (*N*=7) of the studies used a mixed-methods approach. Detailed information of each study is summarized in Additional file 3.

**Table 3:** Characteristics of studies included in the review

	Number of studies	Percentage
**Summary by study region**		
Sub-Saharan Africa	17	33%
East Asia & Pacific	16	31%
Europe & Central Asia	3	6%
Latin America & the Caribbean	1	2%
Middle East & North Africa	5	10%
South Asia	11	21%
**Summary by study settings**		
Primary care facilities	13	25%
Secondary hospitals	4	8%
Tertiary hospitals	39	75%
Community	4	8%
**Summary by intervention population**		
Physicians only	25	48%
Multiple providers	22	42%
Users only	2	4%
Providers and users	3	6%
**Summary by intervention functions in the Behavior Change Wheel**		
Education	27	52%
Enablement	35	67%
Training	16	31%
Environment restructuring	12	23%
Persuasion	4	8%
Restriction	31	60%
Modeling	1	2%
**Summary by the number of intervention functions in the Behavior Change Wheel**		
Single component	11	21%
Two components	15	29%
Three components	18	35%
Multiple components	8	15%
**Summary by study design**		
Cross-sectional	15	29%
Pre-post	17	33%
Quasi experimental	1	2%
Randomized control trial	1	2%
Mixed-methods	7	13%
Case-control	1	2%
Case study	1	2%
Narrative description	2	4%

Among quantitative studies that employed a non-randomized control trial design, most did not report complete outcome data or adjust for confounders in the analysis. Most studies that applied descriptive cross-sectional methods used sampling strategies inappropriate to address the research question. The nonresponse bias was also high across these studies. While most qualitative studies had relatively fewer biases, only one study had comparatively lower quality than the others. In many mixed-methods studies, the divergences and inconsistencies between quantitative and qualitative results were not adequately addressed. Three studies were not assessed using the mixed-methods appraisal tool, since they were narrative descriptions of the implementation process of an intervention or a conceptual model. Additional file 4 provides a breakdown of the quality assessment of each study.

### Barriers and facilitators

We grouped the barriers and facilitators reported in the studies into the five domains and 39 constructs of the CFIR framework and tabulated the frequency with which constructs were addressed (Table 4). We only presented constructs that were addressed in at least five studies to ensure they are common challenges or facilitators shared across LMIC settings. Specific codes for barriers and facilitators based on the CFIR framework of each study are presented in Additional file 5.

**Table 4:** Overview of CFIR constructs that were addressed in studies as barriers or facilitators

CFIR framework constructs		Barriers		Facilitators	
		Number of studies	Specific barriers	Number of studies	Specific facilitators
1	Intervention characteristics				
A	Intervention source			**6**	Local stakeholders were involved in intervention development to ensure ownership, buy-in, and participation.
B	Evidence strength & quality	**9**	Local data on AMR patterns was needed to guide the development of recommendations and guidelines, but it was difficult to obtain reliable AMR data in LMIC settings.	**8**	The intervention (e.g., guidelines and education material) was developed by authoritative and credible sources. Guidelines developed based on local AMR pattern, available resources and needs of implementation facilities.
D	Adaptability			**7**	Intervention material was designed to be incorporated into the local system, adapted to local capacities and priorities, and delivered jointly by local and international team.
G	Design quality & packaging	**6**	Poorly designed interventions, such as few details in the guidelines, insufficient implementation time. Using ineffective approaches to deliver interventions.	**9**	Using interactive and innovative approaches and user-friendly tools to deliver the intervention. Guidelines were needed as way to enforce behavior change.
2	Outer setting				
A	Patient needs & resources	**8**	Patients' needs of antibiotics affected prescribers' decisions on prescribing antibiotics, as they were often pressured by patients to prescribe antibiotics.		
D	External policy & incentives	**12**	It was difficult to enforce antimicrobial stewardship in countries without national policies or guidelines on antibiotic use. Weak enforcement of existing regulations on retailers and nation-wide health facilities. As a result, users could access antibiotics from other sources.	**10**	The interventions were developed in line with national initiatives and priorities. Availability of nation-wide policies and standardized guidelines.
3	Inner setting				
A	Structural characteristics	**22**	The health facilities where interventions were implemented were lacking infrastructure for implementation, such as insufficient laboratory capacity to provide data on AMR patterns and diagnostic results timely, lack of data management system for audit activities, and lack of in-hospital pharmacies. The health facilities did not have a sustainable supply of effective antibiotics. Prescribers often had limited access to diagnostic tests. The health facilities did not have an established governance structure to lead antimicrobial stewardship activities and behavior change interventions. Researchers faced difficulties in working with different levels of administrative systems in tertiary health facilities. High turnover of medical staff in health facilities.	**8**	Creating an environment, in which the participants could carry out the intended behaviors, such as establishing a microbiology laboratory and enhancing the supply of antibiotics.
B	Networks & communications			**8**	The intervention was developed and implemented by an experienced and well-coordinated team of local and international stakeholders with mutual trust. Ensuring good communication among implementers, participants, and other stakeholders.
C	Culture	**7**	Interventions that were developed in a Western context were difficult to implement in other contexts. Disconnect between physicians and other medical staff, such as laboratory technicians and pharmacists. Physicians often resisted accepting suggestions from nurses or pharmacists. A rigid hierarchical structure frequently prohibited junior staff from challenging the prescribing decisions made by senior staff. Tension between doctors and patients during consultations as a barrier to providing adequate antibiotic education for patients.		
E1	Leadership engagement	**7**	Lack of involvement of higher-level leadership and stakeholders in the health facilities. Lack of support from administrative staff.	**21**	Receiving support from higher level stakeholders (e.g., officials from Ministry of Health, health authorities, leaders in health facilities) and administrative staff.
E2	Available resources	**23**	Lack of sustainable financial support for antimicrobial stewardship activities. Shortage of human resources (e.g., microbiologists, pharmacists, and physicians) to implement interventions. Target populations were too busy to perform intervention activities. Lack of technological support to facilitate the implementation of interventions.	**5**	Availability of technology (e.g., digital tools or electronic medical record systems) for managing data and improve the efficiency of the intervention. Leveraging locally available but untapped resources for implementing interventions.
E3	Access to knowledge & information			**9**	Employing training, education, and other promotional strategies helped participants access intervention information and familiarize themselves with intervention activities and content.
4	Characteristics of individuals				
A	Knowledge & beliefs about the intervention	**8**	Target populations often lacked awareness of the ongoing behavior change interventions and activities. In some cases, they were concerned about the effectiveness of the intervention or unfamiliar with the intervention content.	**7**	Participants acknowledged the intervention to be important and useful, and the intervention further promoted their awareness of AMR.
C	Individual stage of change	**11**	Target populations sometimes were reluctant or even resisted to change their routine practice, because participants were skeptical about the effectiveness of the intervention. In some cases, participants had already established perceptions around “best practices” for treatment.		
E	Other personal attributes	**6**	Prescribers often lacked motivation for changing their prescribing practice. Some commented on concerns with complaints from patients and reduction in salary.		
5	Process				
B	Engaging			**13**	Involving a multidisciplinary team of physicians, clinicians, and nurses.
B2	Formally appointed internal implementation leaders			**8**	A dedicated focal person for coordinating antimicrobial stewardship activities (e.g., performing auditing) and support hospital administration was needed. In many studies, pharmacists often took the role as the focal person.
D	Reflecting & evaluating	**5**	Lack of standard indicators for evaluating the effectiveness of behavior change interventions. Lack of systems that continue to collect data for monitoring and evaluating the effectiveness of the interventions.	**5**	Regular monitoring and evaluation of the program using robust methods helped program managers to identify gaps and areas for improvement.

#### Domain 1: intervention characteristics

Intervention source, which refers to the perception of key stakeholders about whether the intervention is externally or internally developed, was reported in six studies as facilitators [36–41]. In these studies, local stakeholders were involved in the development, implementation, or evaluation of interventions, which ensured their ownership and buy-in.

A total of 17 studies addressed evidence strength and quality as a factor for implementing behavior change interventions to promote rational antibiotic use. One shared challenge was the lack of reliable data on AMR patterns in LMIC settings, which is crucial to the development of localized recommendations and guidelines for an antibiotic prescription [39, 42–49]. Eight studies reported that stakeholders and target populations were more receptive to recommendations and guidelines that were developed by authoritative and credible sources or based on localized and reliable evidence [41, 43, 50–55].

Seven studies commented on the adaptability of an intervention that refers to the degree to which interventions were tailored to the local needs [37–40, 52, 54, 56]. Specifically, interventions that were designed to be embedded into routine practice, adapted to the local capacities and priorities, and delivered jointly by local and international teams were more likely to be accepted by target populations.

The design quality and packaging of interventions were reported in nine studies as a facilitator, as using innovative approaches or user-friendly tools to deliver interventions improved the intervention uptake [38, 49, 51, 53, 57–61]. For example, concise guidelines that clearly outlined the appropriate antibiotics for common infections and interactive training sessions were reported to be more receptive by prescribers. Furthermore, six studies reported that poorly designed interventions (such as lack of details in guidelines for antibiotic prescription and insufficient implementation time) or using ineffective approaches to deliver them (such as the development of guidelines without dissemination strategies) were barriers to achieving intended behavior change outcomes [36, 46, 50, 62–64].

#### Domain 2: outer setting

Eight studies reported patient needs and resources as a barrier to changing prescribing practice, as participants were often pressured by patients to prescribe antibiotics, despite the ongoing interventions [39, 48, 49, 51, 60, 62, 65, 66]. External policy and incentives were addressed in a total of 22 studies. Specifically, 12 studies found that it was difficult to promote appropriate behavior among prescribers and users in countries without national policies or guidelines for antibiotic use or those with weak enforcement of existing regulations [39, 40, 43, 48, 52, 63, 67–72]. In contrast, interventions that were developed in line with national AMR initiatives and the availability of national policies and guidelines facilitated the implementation of interventions to improve rational antibiotic use [38, 40, 46, 48, 52, 68, 70, 73–75].

#### Domaine 3: inner setting

A total of 30 studies reported barriers and facilitators in the structural characteristics construct, which refers to the organizational environment where interventions are conducted. Infrastructure constraints included insufficient laboratory capacity to provide data on AMR patterns and diagnostic results timely, lack of data management technology for auditing antimicrobial stewardship activities, lack of in-hospital pharmacies, and inadequate supply of high-quality and effective antibiotics [37, 39–41, 43, 44, 46–48, 52, 66–70, 73, 74, 76–78]. Furthermore, several studies reported that health facilities in LMICs did not have an established governance structure to lead antimicrobial stewardship programs [46, 48, 63, 67], and that high turnover of medical staff and bureaucracy within hospitals prevented the successful implementation of antimicrobial stewardship programs [40, 68]. On the contrary, improving the infrastructure of facilities where interventions were conducted, such as establishing microbiology laboratories and enhancing the supply of antibiotics, facilitated prescribers to make evidence-based decisions on treating patients with appropriate antibiotics [36, 43, 48, 50, 56, 60, 62, 68].

Networks and communications were reported in eight studies as a facilitator [37, 43, 47, 50, 56, 62, 79, 80]. Successful interventions were often developed and implemented by an experienced and well-coordinated team of local and international stakeholders. Various measures were taken to ensure good communication among implementers, participants, and higher-level stakeholders. For example, stakeholders held regular meetings that involved providing project updates and collaborative decision-making to facilitate project planning, implementation, monitoring, and evaluation, as well as keeping track of the achievement of set goals. Researchers also established platforms for efficient communication between medical staff, thus enabling optimal treatment of patients with antibiotics [43].

The norms and values of an organization, which are represented by the culture construct in the framework, were addressed in seven studies [41, 50, 58, 62, 68, 77, 81]. For example, interventions that were developed in the Western context and based on principles of democracy and teamwork were difficult to implement in organizations with a hierarchy culture [50]. In several studies that evaluated the outcomes of antimicrobial stewardship programs, a rigid hierarchy within institutions and disconnection between physicians and other medical staff prevented prescribers from accepting suggestions for antibiotic choices from junior staff or pharmacists who were responsible for auditing prescribing behaviors [41, 50, 68]. Additionally, tension and distrust between physicians and patients were reported as a barrier to educating patients about rational antibiotic use during medical consultation [81].

Leadership engagement refers to the degree to which leaders are committed to, involved in, and held accountable for the implementation of interventions. In 21 studies, the involvement and support of higher-level stakeholders (such as officials from the Ministry of Health, health authorities, experts, leaders in health facilities) and administrative staff were reported as a common facilitator for the successful implementation of behavior change interventions [37, 39, 40, 44, 48–50, 52, 54, 56, 58, 59, 62, 65, 70, 78–83]. The lack thereof had been documented to impede intervention implementation in seven studies [43, 46, 60, 63, 69, 71, 84].

In 23 studies, the common challenges faced by implementers and researchers were related to resources, including insufficient financial support for sustaining antimicrobial stewardship activities, shortage of human resources (such as microbiologists, pharmacists, and infectious disease specialists), and lack of technological support to facilitate the administration of interventions [38, 42–46, 48–50, 58, 62, 63, 67, 69–71, 73, 75–77, 79, 85, 86]. It was also commonly reported that prescribers participating in ASPs were often too busy to perform intervention activities. On the contrary, the availability of information technology for managing auditing data and patients’ records had reduced the workload of participants and improved the efficiency of managing antimicrobial stewardship programs. Researchers and implementers also leveraged locally available but often untapped resources to ensure that interventions could be sustained [44, 47, 50, 62, 73].

Access to knowledge and information about interventions was recognized as a facilitator in nine studies [41, 43, 51, 53, 55, 61, 65, 80, 83]. Implementers and researchers employed training, education sessions, and other publicity strategies to help participants access intervention information and familiarize with intervention activities and content.

#### Domain 4: characteristics of individuals

Eight studies reported barriers in the “knowledge and beliefs about the intervention” construct. In several studies, the target populations were not aware of the ongoing interventions. Sometimes, participants were concerned about the effectiveness of the intervention or unfamiliar with the intervention content [39, 63, 66, 68, 69, 73, 75, 76]. On the other hand, seven studies documented that participants were more receptive to behavior change interventions when they acknowledge these interventions to be important and useful in improving their awareness of AMR [41, 47, 49, 56, 68, 79, 81].

The individual stage of change was addressed in 11 studies as a barrier to changing prescribing practice [40, 41, 49, 59, 60, 63, 66, 68, 69, 71, 75]. Prescribers sometimes were reluctant or even resistant to change their habitual practice, because they were skeptical about the effectiveness of the interventions. In some cases, prescribers had already established perceptions around “best practices” for treatment, which was difficult to change.

Additionally, six studies reported that lack of motivation prevented behavior change of prescribers, as some were concerned about complaints from patients or reduction in salary if they refused to prescribe antibiotics [39, 48, 62, 78, 84, 86].

#### Domain 5: process

The engaging construct was addressed in 13 studies, which acknowledged that effective implementation of behavior change interventions to promote rational antibiotic use required the involvement and collaboration of a multidisciplinary team of medical staff, including physicians, clinicians, nurses, and pharmacists [44, 48, 54, 56, 57, 66, 70, 72, 73, 75, 79, 82, 87]. In eight studies, appointing a dedicated focal person, usually a pharmacist or an infectious disease specialist, for coordinating antimicrobial stewardship activities (such as ward rounds, auditing antibiotic prescribing behavior, and supporting intervention management) was a facilitator [36, 47, 52, 55, 61, 70, 74, 83].

Five studies acknowledged that regular monitoring and evaluation of interventions using robust methods helped implementers to identify gaps and areas for improvement [52, 56, 76, 83, 87]. However, this was difficult to achieve in settings where routine data for monitoring and evaluation was not available. Researchers and implementers were also concerned about the validity of outcome indicators being used for assessing the effectiveness of behavior change interventions [42, 44, 69, 79, 85].

### Construct relationships

**Fig. 2:**
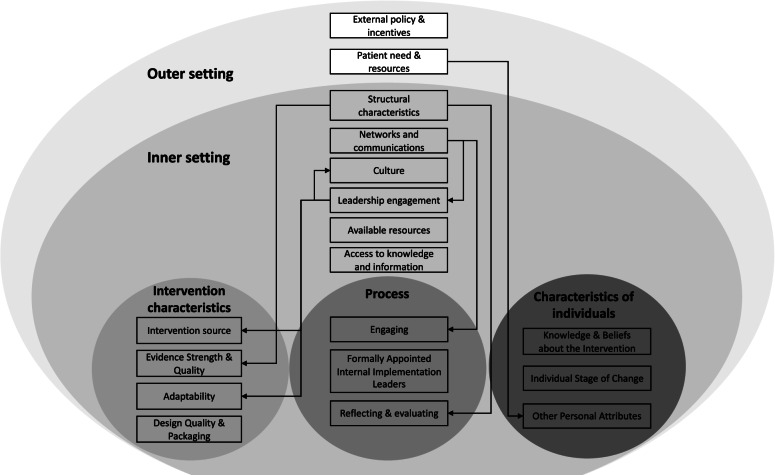
A map of the relationships between the constructs that were addressed in studies as barriers or facilitators

We found that several factors influencing the implementation of the behavior change interventions were interconnected as shown in Fig. 2. For example, insufficient laboratory capacity to provide data on AMR patterns (structural characteristics) hindered the development of context-specific guidelines on antibiotic use, which was commonly reported as a challenge in ASPs (evidence strength and quality) [39, 42, 43]. The lack of data management systems for auditing prescribing behaviors and antibiotic use (structural characteristics) led to insufficient capacity for monitoring and evaluating behavior change interventions (reflecting and evaluating) [42, 69, 79]. Another example is that engaging local stakeholders who were responsible for policymaking or program decisions (leadership engagement) facilitated the development of interventions to be incorporated into routine practice (adaptability), aligned with local priorities, and better fitted into the organizational culture (culture) [37–40]. It also helped to build ownership and buy-in from stakeholders (intervention source) and facilitate policy scale-up [36–39, 41, 88]. The engagement with stakeholders, as well as between intervention participants, was usually facilitated by good communication (networks and communications) [37, 43, 47, 50, 81]. Additionally, studies reported that patients sometimes pressured physicians to prescribe antibiotics (patients’ needs and resources), which might contribute to low motivation for changing prescribing behavior among prescribers (other personal attributes) [60, 62, 65, 81].

## Discussion

By using a comprehensive implementation science framework, our review systematically synthesizes barriers and facilitators of implementing behavior change interventions that aimed to improve appropriate antibiotic use in LMICs across the five domains of the CFIR framework. The most commonly reported factors influencing implementation were found in the inner setting domain, particularly related to the available resources and the structural characteristics of organizations where interventions were conducted. Barriers related to the external policy environment (outer setting domain) and individual characteristics of target populations were also common, as well as facilitators related to intervention characteristics and process. Additionally, we identified relationships between constructs and across domains, which shows the complex influences at play during implementation. Although our review included interventions targeting both prescribers and users, we found that most of these interventions were designed for prescribers practicing at tertiary health facilities.

The external policy environment, particularly the existence of national initiatives and guidelines on antibiotic use, was commonly documented to influence the implementation of antimicrobial stewardship interventions for prescribers. Setting policy priorities or launching national initiatives for tackling AMR would create a supportive policy environment that often bolsters the engagement of high-level stakeholders and prompts the allocation of resources that are directed towards actions to generate the optimal effect [89, 90]. Lack thereof often contributes to the shortage of sustainable financial, human, and technological resources—the most reported implementation barrier in our review and other similar reviews of implementing health interventions in LMICs [91, 92]. Since 2015 when the WHO’s GAP for AMR was endorsed by 196 member states, the majority of countries have published national action plans. However, few countries have these action plans developed to reflect the objectives of the GAP with an operational plan and monitoring arrangements, and even fewer have identified funding sources and been implemented [93]. This lack of political momentum in many countries has led to the inadequate investment of domestic resources and a fragmented strategy towards AMR that is often predominated by donor-driven projects [94]. International donors have long supported the efforts to tackle AMR issues in LMICs by providing funding and technical support. However, investment from international donors is often short-term and project-based, and a large proportion of international investment has been focusing on pharmaceutical solutions [95, 96]. Furthermore, international attention on AMR evolves in waves, given the many other competing policy priorities in global health [97]. Despite AMR being a persistent and accelerating problem, global attention (measured by the number of reports and international funding) has decreased since 2017 with interest further waning and resources diverted due to the demands of the COVID-19 pandemic [97, 98].

In addition, we found that the implementation of behavior change interventions was often impeded by infrastructure limitations in the organizations where interventions were conducted. Consistent with a previous review of ASPs in LMICs, our findings showed that lack of laboratory and diagnostic tools, surveillance of AMR and antibiotic use, and reliable supply of antibiotics was common in health facilities in LMICs [27]. As a result, it was difficult to develop context-specific protocols and guidelines for antibiotic use without accurate information on AMR patterns, which was recognized as a barrier for developing high-quality interventions in the reviewed studies. Correct identification of pathogens and susceptibility is the foundation for evidence-based clinical practice, but without the basic infrastructure to provide this essential information, prescribers had to rely on their experiences to make clinical decisions, even if guidelines and protocols suggested otherwise. Additionally, challenges in access to effective generic antibiotics are salient in both high- and LMICs. Because of economic reasons, such as high registration cost and low return on investment, these antibiotics are often available on the market in only a few countries [99]. As a result, prescribers often had to choose less optimal but broad-spectrum antibiotics, which might lead to the selection of resistance [100, 101].

The common implementation barriers and facilitators that were identified in the intervention characteristics and inner setting domains indicated that contextualizing behavior change interventions (developed by local stakeholders based on reliable and context-specific evidence) so that they addressed local needs and priorities, fitted into routine practice, and were delivered using culturally sensitive methods was key to the successful implementation of behavior change interventions. Our review highlighted the importance of engaging local stakeholders using a participatory approach. Since some interventions tackling AMR were developed and proved to be effective in high-income countries, in the reviewed studies, researchers engaged local leadership (such as program decision-makers and administrators) not only to facilitate buy-in from stakeholders in the implementation process but also as a strategy to contextualize interventions. This is consistent with previous implementation research that engaging local stakeholders who are responsible for making program or policy decisions in developing, adapting, implementing, and evaluating these interventions has been widely used to build local ownership and improve cultural sensitivity, receptibility, and feasibility of interventions in other health interventions [102]. Our findings also supported previous studies that antimicrobial stewardship interventions that embedded in the existing organizational infrastructure of health facilities would be sustainable and more likely to be scaled up [88, 103]. In practice, researchers commonly maintained good communication with stakeholders during the implementation process, such as holding regular meetings for updating the progress or conducting interviews or workshops to understand their interests and needs. Participatory approaches, which involve stakeholders in shared decision-making, were also commonly adopted in the reviewed studies and current literature on implementation research [50, 59, 82, 104, 105]. In addition to decision-makers, we found that involving target populations in the development of behavior change interventions was helpful in identifying participants’ needs and concerns, raising their awareness and knowledge about the interventions, and ensuring that the developed tools and protocols were user-friendly and could be incorporated into routine practice, hence solving the implementation challenges identified in the reviewed studies.

We identified a gap in current practice to address inappropriate antibiotic use in LMICs. Consistent with other reviews, we found that most interventions targeted prescribers who are practicing at tertiary hospitals, while there is a dearth of interventions focusing on users and the wider community [23, 24, 26]. However, participants of ASPs reported that they were pressured by patients to prescribe antibiotics or concerned with complaints from patients if they refused to prescribe antibiotics [60, 78, 84]. It is also well documented that patients could access antibiotics from sources in communities, such as primary healthcare clinics or informal sellers [48, 49, 106]. Hence, tackling AMR through behavior change interventions should include both the supply- and demand-side.

### Implications and recommendations for policy and practice

By giving an overview of implementation barriers and facilitators through an implementation science lens, our review suggests that facilitating the successful implementation of behavior change interventions for improving antibiotic use in LMICs needs a comprehensive approach to resolve contextual, organizational, and process-oriented challenges. Therefore, we propose the following recommendations for policy and practice, as these are the areas that need to be addressed so that future behavior change interventions for antibiotic providers and users in LMICs can be effectively implemented (Table 5).

**Table 5:** Summary of recommendations for policy and practice

	Recommendations
Policy level	Strengthening political commitment to combating AMR and prioritizing the issue to increase investment of domestic resources and prompt formulation and implementation of more sustainable strategies on AMR.
Organizational level	Improving the infrastructure of health facilities that allow prescribers to make evidenced-based decisions on treating patients with antibiotics. Specific strategies include the following: • Building laboratories to facilitate diagnosis • Enhancing surveillance of AMR and antibiotic use for developing context-specific guidelines and strengthening evaluation of AMR interventions • Securing supply of antibiotics to ensure availability of effective antibiotics
Implementation level	Engaging local stakeholders using a participatory approach to facilitate buy-in and contextualizing interventions, ensuring that the interventions address local needs and priorities, fit into routine practice, and are delivered using culturally sensitive methods. Specific strategies include: • Involving local leadership and decision-makers in developing, adapting, implementing, and evaluating interventions. • Involving target population in the development and planning of interventions to ensure that the developed tools and protocols can be incorporated into routine practice.

First, to ensure sustainable efforts to address AMR issues in LMICs, increasing investment of domestic resources is needed. With the waning of international funding and attention for AMR, particularly in the midst of the COVID-19 pandemic, strengthening high-level political commitment to tackling AMR, and prioritizing the issue at the policy level in LMICs is the precedent determinant for mobilizing domestic resources to support local interventions and programs, such as strengthening infrastructure for diagnosis and surveillance, training medical and laboratory staff, and upgrading technology for case management. It will also facilitate collaboration across a wide range of sectors and prompt the formulation and implementation of a more sustainable strategy on AMR.

Second, it is crucial to improve the infrastructure of health facilities where antimicrobial stewardship activities are implemented, such as establishing laboratories, enhancing surveillance, and securing the supply of effective antibiotics. This will strengthen the evidence base that informs the development of context-specific guidelines for antibiotic use and create an environment that allows prescribers to practice intended prescribing behaviors as instructed in guidelines or training.

Finally, contextualizing behavior change interventions to promote appropriate antibiotic use can be achieved through engaging local stakeholders using a participatory approach. Specifically, researchers and program implementers can involve local leadership and decision-makers, as well as target populations, in developing, adapting, implementing, and evaluating interventions so that these interventions address local needs and priorities, fit into routine practice, and are delivered using culturally sensitive methods.

### Strengths and limitations

To our knowledge, this review is the first to provide an overview of the barriers and facilitators of implementing behavior change interventions that aim to promote rational antibiotic use in LMICs. Our review was conducted using rigorous methods and guided by a framework developed from the implementation research perspective, which allowed us to address the research question comprehensively and in a systematic way. We also provided insights into the interrelationships between the commonly reported implementation barriers and facilitators, as well as the underlying factors contributing to the implementation challenges in LMICs. Our review recommends a comprehensive approach to addressing the challenges in AMR containment efforts in LMICs and calls for commitment and immediate actions from high-level policy makers. The findings are also helpful for researchers and implementers to directly assess the barriers posed by context when developing similar interventions and encourage monitoring of these barriers and facilitators during intervention implementation in future studies.

However, it has the following limitations. First, our review did not include gray literature or articles not in English or Chinese; thus, we might miss relevant studies. Second, we synthesized commonly reported implementation barriers and facilitators based on the frequency of which was addressed in the reviewed studies without concluding on their relative importance, as we acknowledge that the relative importance of these factors is context-dependent. Third, we were aware that many included studies were not specifically designed to examine implementation barriers and facilitators. Although we aimed to extract implementation barriers and facilitators experienced by researchers, as stated in the inclusion criteria, the reviewed studies sometimes failed to distinguish between actual experiences and speculations. We acknowledge that speculations of the potential implementation barriers and facilitators by authors may not always be the actual ones. Finally, despite reporting on quality appraisal, we did not exclude studies based on the quality to ensure the comprehensiveness of our summary on the topic. Furthermore, all studies were treated equally in the synthesis without given undue weight based on the study quality.

## Conclusion

Guided by the CFIR framework, we gave an overview of the barriers and facilitators of implementing behavior change interventions to promote rational antibiotic use in LMICs through an implementation science lens. Our findings indicate that facilitating the implementation of interventions to improve rational antibiotic use needs comprehensive efforts to address challenges at policy, organizational, and implementation levels. Strengthening political commitment and prioritizing AMR at the policy level will facilitate mobilizing domestic resources to combat AMR and prompt the development of a sustainable strategy for AMR. At the organizational level, improving the infrastructure of health facilities where antimicrobial stewardship activities are implemented is needed to strengthen the evidence base for developing context-specific guidelines for antibiotic use and create an environment that allows prescribers to practice appropriate prescribing. Engaging local stakeholders is a useful strategy to improve their buy-in throughout the implementation process and facilitates contextualizing interventions.

## Supplementary Information

**Additional file 1.** Search strategy.

**Additional file 2.** Code book.

**Additional file 3.** Summary table of included studies.

**Additional file 4.** Summary of quality appraisal.

**Additional file 5.** Summary of specific codes for barriers and facilitators based on the CFIR framework of the included studies.

**Additional file 6.** PRISMA checklist.

## Data Availability

All data generated or analyzed during this study are included in this published article [and its supplementary information files].

## Abbreviations

AMR: Antimicrobial resistance
ASPs: Antimicrobial stewardship programs
CFIR: Consolidated Framework for Implementation Research
GAP: Global Action Plan
LMICs: Low- and middle-income countries
WHO: World Health Organization
