# Supplementary material for: Barriers and facilitators of implementing interventions to improve appropriate antibiotic use in low- and middle-income countries: a systematic review based on the Consolidated Framework for Implementation Research
Source: Implement Sci. 2022 May 12;17:30. doi: 10.1186/s13012-022-01209-4 (PMC9096759; doi:10.1186/s13012-022-01209-4)
Supplement: Supplementary file 1 — Additional file 1. Search strategy. [file 13012_2022_1209_MOESM1_ESM.docx]

**Additional file 1. Search strategy**

Search strategy for MEDLINE

| 1 | antimicrobial stewardship/ or antibiotic stewardship/ | 1973 |
| --- | --- | --- |
| 2 | (AMS or antimicrobial stewardship or antibiotic stewardship).tw,kf. | 12729 |
| 3 | Anti-Bacterial Agents/ | 347657 |
| 4 | (anti-bacterial* or antibacterial* or anti-mycobacterial* or antimycobacterial* or antibiotic* or antimicrobial* or anti-infective or antimicrobial agent* or anti-microbial agent* or antimicrobial resistance or anti-microbial resistance or antibiotic resistance or AMR).tw,kf. | 553196 |
| 5 | Drug prescription/ or drug utilization/ | 45717 |
| 6 | (prescribe* or prescription or "appropriate adj2 use" or "appropriate adj2 utiliz*" or "drug use" or "medicine use" or "rational adj2 use" or consumption).mp. | 616883 |
| 7 | Health plan implementation/ or program evaluation/ | 70153 |
| 8 | (intervention*or program* or implement* or campaign or evaluat* or assess* or pilot or impact).tw,kf. | 6965074 |
| 9 | Guideline adherence/ or Health promotion/ or health education/ | 163502 |
| 10 | (stewardship or behavior or behavioral or behaviour or behavioural or behavior change or behaviour change or supervision or audit or feedback or reminder* or regulat* or guideline* or train* or educat*or persuasion or incentiv* or coercion or restrict* or environmental restructur* or modeling or enablement or awareness or communit* or school* or communicat* or mass media or booklet* or poster* or pamphlet* or leaflet* or advocacy or information or outreach or subsid* or reward* or ban or prohibit* or television or TV or social media or facebook or twitter or text messag*or sms).tw,kf. | 6817233 |
| 11 | Developing countries/ | 76448 |
| 12 | (afghanistan or albania or algeria or american samoa or angola or argentina or armenia or armenian or azerbaijan or bangladesh or republic of belarus or belarus or byelarus or belorussia or byelorussian or belize or british honduras or benin or dahomey or bhutan or bolivia or "bosnia and herzegovina" or bosnia or herzegovina or botswana or bechuanaland or brazil or brasil or bulgaria or burkina faso or burkina fasso or upper volta or burundi or urundi or cabo verde or cape verde or cambodia or kampuchea or khmer republic or cameroon or cameron or cameroun or central african republic or ubangi shari or chad or china or colombia or comoros or comoro islands or iles comores or mayotte or democratic republic of the congo or democratic republic congo or congo or zaire or costa rica or "cote d’ivoire" or "cote d’ ivoire" or cote divoire or cote d ivoire or ivory coast or cuba or djibouti or french somaliland or dominica or dominican republic or ecuador or egypt or united arab republic or el salvador or equatorial guinea or spanish guinea or eritrea or eswatini or swaziland or ethiopia or fiji or gabon or gabonese republic or gambia or "georgia (republic)" or georgian or ghana or gold coast or grenada or guatemala or guinea or guinea bissau or guyana or british guiana or haiti or hispaniola or honduras or india or indonesia or timor or iran or iraq or jamaica or jordan or kazakhstan or kazakh or kenya or "democratic people’s republic of korea" or north korea or kosovo or kyrgyzstan or kirghizia or kirgizstan or kyrgyz republic or kirghiz or laos or lao pdr or "lao people's democratic republic" or lebanon or lebanese republic or lesotho or basutoland or liberia or libya or libyan arab jamahiriya or republic of north macedonia or macedonia or madagascar or malagasy republic or malawi or nyasaland or malaysia or malay federation or malaya federation or maldives or indian ocean islands or indian ocean or mali or micronesia or federated states of micronesia or kiribati or marshall islands or tuvalu or mauritania or mexico or moldova or moldovian or mongolia or montenegro or morocco or ifni or mozambique or portuguese east africa or myanmar or burma or namibia or nepal or netherlands antilles or nicaragua or niger or nigeria or muscat or pakistan or papua new guinea or new guinea or paraguay or peru or philippines or philipines or phillipines or phillippines or russia or russian federation or ussr or soviet union or union of soviet socialist republics or rwanda or ruanda or samoa or pacific islands or polynesia or samoan islands or navigator island or navigator islands or "sao tome and principe" or senegal or serbia or sierra leone or melanesia or solomon island or solomon islands or norfolk island or norfolk islands or somalia or south africa or south sudan or sri lanka or ceylon or saint lucia or "st. lucia" or "saint vincent and the grenadines" or saint vincent or "st. vincent" or grenadines or sudan or suriname or surinam or dutch guiana or netherlands guiana or syria or syrian arab republic or tajikistan or tadjikistan or tadzhikistan or tadzhik or tanzania or tanganyika or thailand or siam or timor leste or east timor or togo or togolese republic or tonga or tunisia or turkey or turkmenistan or turkmen or uganda or ukraine or uzbekistan or uzbek or vanuatu or new hebrides or venezuela or vietnam or viet nam or middle east or west bank or gaza or palestine or yemen or yugoslavia or zambia or Zimbabwe).mp. | 1674137 |
| 13 | (LMIC* or low income countr* or middle income countr* or developing countr*).tw,kf. | 119171 |
| 14 | (low adj3 middle adj3 countr*).tw,kf. | 20794 |
| 15 | (1 or 2) and (7 or 8) | 6449 |
| 16 | (3 or 4) and (5 or 6) and (7 or 8) and (9 or 10) | 7104 |
| 17 | 11 or 12 or 13 or 14 | 1801922 |
| 18 | 15 or 16 | 11941 |
| 19 | 17 and 18 | **2005** |
